# Knockdown of SLC41A1 magnesium transporter promotes mineralization and attenuates magnesium inhibition during osteogenesis of mesenchymal stromal cells

**DOI:** 10.1186/s13287-017-0497-2

**Published:** 2017-02-21

**Authors:** Yu-Tzu Tsao, Ya-Yi Shih, Yu-An Liu, Yi-Shiuan Liu, Oscar K. Lee

**Affiliations:** 1 Institute of Clinical Medicine, National Yang-Ming University, Taipei, 11221 Taiwan; 2 Division of Nephrology, Department of Medicine, Taoyuan General Hospital, Ministry of Health and Welfare, Taoyuan, 33004 Taiwan; 3 Stem Cell Research Center, National Yang-Ming University, Rm. 825, Chih-Teh Building, No.322, Sec.2, Shih-Pai Rd, Taipei, 11221 Taiwan; 4 Taipei City Hospital, 145 Zhengzhou Road, Taipei, 10341 Taiwan; 5 Department of Medical Research, Taipei Veterans General Hospital, Taipei, 11217 Taiwan

**Keywords:** Mineralization, Magnesium transporter, SLC41A1, Mesenchymal stromal cells, Osteogenic differentiation

## Abstract

**Background:**

Magnesium is essential for numerous physiological functions. Magnesium exists mostly in bone and the amount is dynamically regulated by skeletal remodeling. Accelerating bone mass loss occurs when magnesium intake is insufficient; whereas high magnesium could lead to mineralization defects. However, the underlying magnesium regulatory mechanisms remain elusive. In the present study, we investigated the effects of high extracellular magnesium concentration on osteogenic differentiation of mesenchymal stromal/stem cells (MSCs) and the role of magnesium transporter SLC41A1 in the mineralization process.

**Methods:**

Murine MSCs derived from the bone marrow of BALB/c mouse or commercially purchased human MSCs were treated with osteogenic induction medium containing 5.8 mM magnesium chloride and the osteogenic differentiation efficiency was compared with that of MSCs in normal differentiation medium containing 0.8 mM magnesium chloride by cell morphology, gene expression profile of osteogenic markers, and Alizarin Red staining. *Slc41a1* gene knockdown in MSCs was performed by siRNA transfection using Lipofectamine RNAiMAX, and the differentiation efficiency of siRNA-treated MSCs was also assessed.

**Results:**

High concentration of extracellular magnesium ion inhibited mineralization during osteogenic differentiation of MSCs. Early osteogenic marker genes including osterix, alkaline phosphatase, and type I collagen were significantly downregulated in MSCs under high concentration of magnesium, whereas late marker genes such as osteopontin, osteocalcin, and bone morphogenetic protein 2 were upregulated with statistical significance compared with those in normal differentiation medium containing 0.8 mM magnesium. siRNA treatment targeting SLC41A1 magnesium transporter, a member of the solute carrier family with a predominant Mg^2+^ efflux system, accelerated the mineralization process and ameliorated the inhibition of mineralization caused by high concentration of magnesium. High concentration of magnesium significantly upregulated *Dkk1* gene expression and the upregulation was attenuated after the *Slc41a1* gene was knocked down. Immunofluorescent staining showed that *Slc41a1* gene knockdown promoted the translocation of phosphorylated β-catenin into nuclei. In addition, secreted MGP protein was elevated after *Slc41a1* was knocked down.

**Conclusions:**

High concentration of extracellular magnesium modulates gene expression of MSCs during osteogenic differentiation and inhibits the mineralization process. Additionally, we identified magnesium transporter SLC41A1 that regulates the interaction of magnesium and MSCs during osteogenic differentiation. Wnt signaling is suggested to be involved in SLC41A1-mediated regulation. Tissue-specific SLC41A1 could be a potential treatment for bone mass loss; in addition, caution should be taken regarding the role of magnesium in osteoporosis and the design of magnesium alloys for implantation.

**Electronic supplementary material:**

The online version of this article (doi:10.1186/s13287-017-0497-2) contains supplementary material, which is available to authorized users.

## Background

Bone tissue is formed by osteoblasts, maintained by osteocytes, and broken down by osteoclasts. Physiologically, bone is constantly remodeled coordinately between bone resorption by osteoclasts and bone formation by osteoblasts [1]. Osteoblasts sustain bone mass by secreting proteins which form extracellular matrix that reinforces the strength of bone after calcium deposition [2]. Although extracellular calcification in bone cells has a positive effect for healthy bone, the ectopic calcification that occurs in soft tissues may have severe clinical consequences when localized to organs such as the arteries and kidneys [3]. However, it is still not well known how cells give rise to minerals and what the underlying regulatory mechanisms are.

Magnesium, the second most abundant intracellular free cation and the fourth most abundant metal ion in the body [4], is known for numerous physiological functions, such as enzymatic activation and Mg–ATP complex formation for energy production [5–7]. Magnesium has also been shown to inhibit Wnt/β-catenin activity and reverse calcification of vascular smooth muscle cells [8]. In many of the cellular functions in which magnesium is involved, it acts as a physiological calcium antagonist; moreover, both magnesium and calcium play vital roles in bone metabolism [9, 10], and therefore a balance between calcium and magnesium is important in bone physiology [11]. In the human body, magnesium exists mostly in bone, either on the surface of hydroxyapatite (HAP), which is a major component of bone and teeth, or in the hydration shell around the HAP crystal [12]. Because bone serves as the reservoir for magnesium, the amount of magnesium is regulated dynamically by skeletal remodeling during bone resorption and formation for maintaining proper physiological function [13].

The very limited Mg^2+^ gradient across the cell membrane indicates that there is a tight regulation for Mg^2+^ homeostasis [14]. Several magnesium transporters have so far been identified and suggested to cooperate with each other for this tight regulation [15], yet few of them have been investigated at the molecular level. Among these transporters, solute carrier family 41 member 1 (SLC41A1) has been identified recently as a Na^+^/Mg^2+^ exchanger with a predominant Mg^2+^ efflux system containing the N terminus involved in Mg^2+^ sensing and protein kinase activities [16]. SLC41A1 has been linked with several serious human illnesses such as Parkinson’s disease, preeclampsia, and nephronophthisis-like phenotypes. Moreover, SLC41A1 expression is correlated positively with the level of serum magnesium in head and neck cancer patients under chemotherapy [17]. Because serum magnesium is associated with bone mineral density and calcification, we reason that SLC41A1 may play a role in magnesium regulation for bone metabolism [11, 18]. Additionally, *Slc41a1* is upregulated in some organs when mice are on a low-magnesium-containing diet [19, 20]. Together with the association between *Slc41a1* expression and the level of serum magnesium in mice during exercise [21], we hypothesize that SLC41A1 plays a role in magnesium homeostasis during osteogenic differentiation of MSCs.

Osteoporosis is a progressive disease resulting from an imbalance between bone deposition and resorption. Accelerating bone mass loss occurs in postmenopause animals when their magnesium intake is insufficient, and increased magnesium intake alleviates the osteoporotic symptoms [22]. On the other hand, high magnesium concentration leads to mineralization defects possibly due to magnesium substitution for calcium in the HAP structure [23]. It is reported that slow release of magnesium from scaffolds could contribute to bone regeneration in vivo [24, 25]; whereas a hyperphysiological level of magnesium concentration inhibits extracellular matrix formation and supports chondrocyte proliferation [26]. Therefore, it is essential to understand the regulatory mechanisms in which magnesium is involved in the promotion of mineralization as well as osteoblast generation. Mesenchymal stromal cells (MSCs) possess promising potential in clinical application due to their immunomodulatory effects and the ability to give rise to various mature progenies, such as osteoblasts, adipocytes, and chondrocytes [27, 28]. In the present study, we investigate the effect of high extracellular magnesium concentration on osteogenic differentiation of MSCs and the role of magnesium transporter SLC41A1 in the mineralization process during osteogenic differentiation.

## Methods

### Cell maintenance and expansion

Mouse bone marrow-derived MSCs (mMSCs) were obtained from the femoral and tibial bone marrow of a 7–8-week-old male Balb/c mouse purchased from National Laboratory Animal Center (Taipei, Taiwan) as described previously [29]. The protocols were approved by the Taipei Veterans General Hospital Institutional Animal Care and Use Committee (IACUC 2013-048). All studies involving animals were in accordance with appropriate guidelines. The isolated cells were characterized by the surface markers using flow cytometry and assessed by the osteogenic, adipogenic, as well as chondrogenic differentiation assays before being further used for the study. Human MSCs (hMSCs) were purchased from Cell Applications, Inc. (catalog/lot number 492-05a/2694; San Diego, CA, USA). This specific population of MSCs was tested for multilineage differentiation potential before being further used for the study. Cells from the 12th–15th passages used for the present study have been confirmed for their stemness prior to the experiments by checking the expression of surface markers using flow cytometry, and for their hepatogenic, osteogenic, as well as adipogenic differentiation potential. Protocols used for maintenance and expansion of mMSCs and hMSCs were described previously [30].

### Osteogenic differentiation of MSCs

The basal medium used for osteogenic induction was high-glucose Dulbecco's modified Eagle's medium (HG-DMEM) for mMSCs and Iscove's modified Dulbecco's medium (IMDM) for hMSCs supplemented with the induction factors: 0.1 μM dexamethasone (Sigma-Aldrich), 10 mM β-glycerol phosphate (βGP; Sigma-Aldrich), and 0.2 mM ascorbic acid (ASA; Sigma-Aldrich) [31]. The cells were seeded at 4000 cells/cm^2^ and were maintained in culture medium (low-glucose DMEM supplemented with 10% FBS) for 24 hours before the induction. The induction medium was changed twice a week.

DMEM and IMDM contain 0.8 mM magnesium ion, and therefore 0.8 mM magnesium concentration is defined as normal magnesium concentration. The serum magnesium level in patients with renal disease is around 1 mM [32] and a serum magnesium level around 5 mM is associated with profound muscle weakness; on the other hand, physiological magnesium concentrations in soft tissue and bone are 8.5 and 43.2 mmol/kg wet weight respectively [15]. Therefore, for the induction medium with high concentration of magnesium, DMEM and IMDM were supplemented with 5 mM MgCl_2_ (Sigma-Aldrich) and we defined 5.8 mM MgCl_2_ as the experimental group of high magnesium concentration. A 1 M MgCl_2_ stock solution was prepared in double-distilled water (ddH_2_O) to make the final concentration of magnesium 5.8 mM.

### Quantitative real-time polymerase chain reaction

Total RNA was extracted by RNeasy Mini Kit (QIAGEN, Hilden, Germany) and reverse-transcribed to complementary DNA by MMLV High Performance Reverse Transcriptase according to the manufacturer’s instructions (EPICENTRE, Madison, WI, USA). Quantitative real-time PCR (qPCR) was performed with TaqMan® Fast Universal PCR Master Mix (2×) by the Step One plus real-time PCR system (Applied Biosystems, Foster City, CA, USA) to determine the relative gene expression profiles. Primers used for qPCR are presented in Additional file 1: Table S1 with ribosomal protein S18 (*Rps18*) as the endogenous internal control. Relative mRNA expression was calculated by the difference of ΔC_t_ (ΔΔC_t_) of the target gene from the group without induction (day 0) and under osteogenic induction. The relative value RQ of gene expression was defined as 2^–ΔΔCt^ *.*

### Alizarin Red S staining and MGP detection

For Alizarin Red staining, after washing in ddH_2_O three times formaldehyde-fixed cells were stained with 2% w/v Alizarin Red S solution (pH 4.1) (Sigma-Aldrich) for 20 minutes, followed by further ddH_2_O washing twice. Then 10% cetylpyridinium chloride (CPC; Sigma-Aldrich) was used to extract the dye by incubating the stained cells at 37 °C for 20 minutes. The absorbance at 550 nm was measured for quantification.

For qualitative detection of mouse matrix gla protein (MGP) secreted from mMSCs in condition medium, mMSCs were cultured in 6-cm culture dishes. Fresh induction medium was added into culture dishes 3 days before being collected for the assay. MGP assay was performed by MGP ELISA kit according to the manufacturer’s instruction (MyBioSource, San Diego, CA, USA) and the amount of MGP secretion was normalized by the cell number of the control group.

### siRNA transfection

siRNA targeting *Slc41a1* was transfected into mMSCs according to the manufacturer's instructions. Briefly, 50 nM of siRNA against *Slc41a1* gene (UCGGAGUCAUCAUUGGGUCUCGAAA and UUUCGAGACCCAAUGAUGACUCCGA, Slc41a1MSS294789) was transfected with RNAiMAX transfection reagent (Invitrogen) into mMSCs for 24 hours at 37 °C. Cells were then washed with PBS and cultured in osteogenic induction medium afterward.

### Immunofluorescent staining

Primary antibodies against phosphorylated β-catenin (pSer^33^/pSer^37^; Abcam, UK) and phosphorylated p38 MAPK (pThr^180^/pTyr^182^; Cell Signaling Technology, USA) were used at 1:200 dilution according to the manufacturers’ instructions. Images were taken by Olympus FluoView™ FV10i confocal microscope on the same day using the same settings of laser power and pinhole size.

### Statistical analysis

All data for statistical analysis were derived from at least three independent experiments (indicated as *N*) and three technical repeats (indicated as *n*). Data were presented as mean ± SEM. Paired Student's t test was used to compare data from two groups. *p* < 0.05 was considered statistically different.

## Results

### High concentration of extracellular magnesium inhibited mineralization of mMSCs and hMSCs

**Fig. 1:**
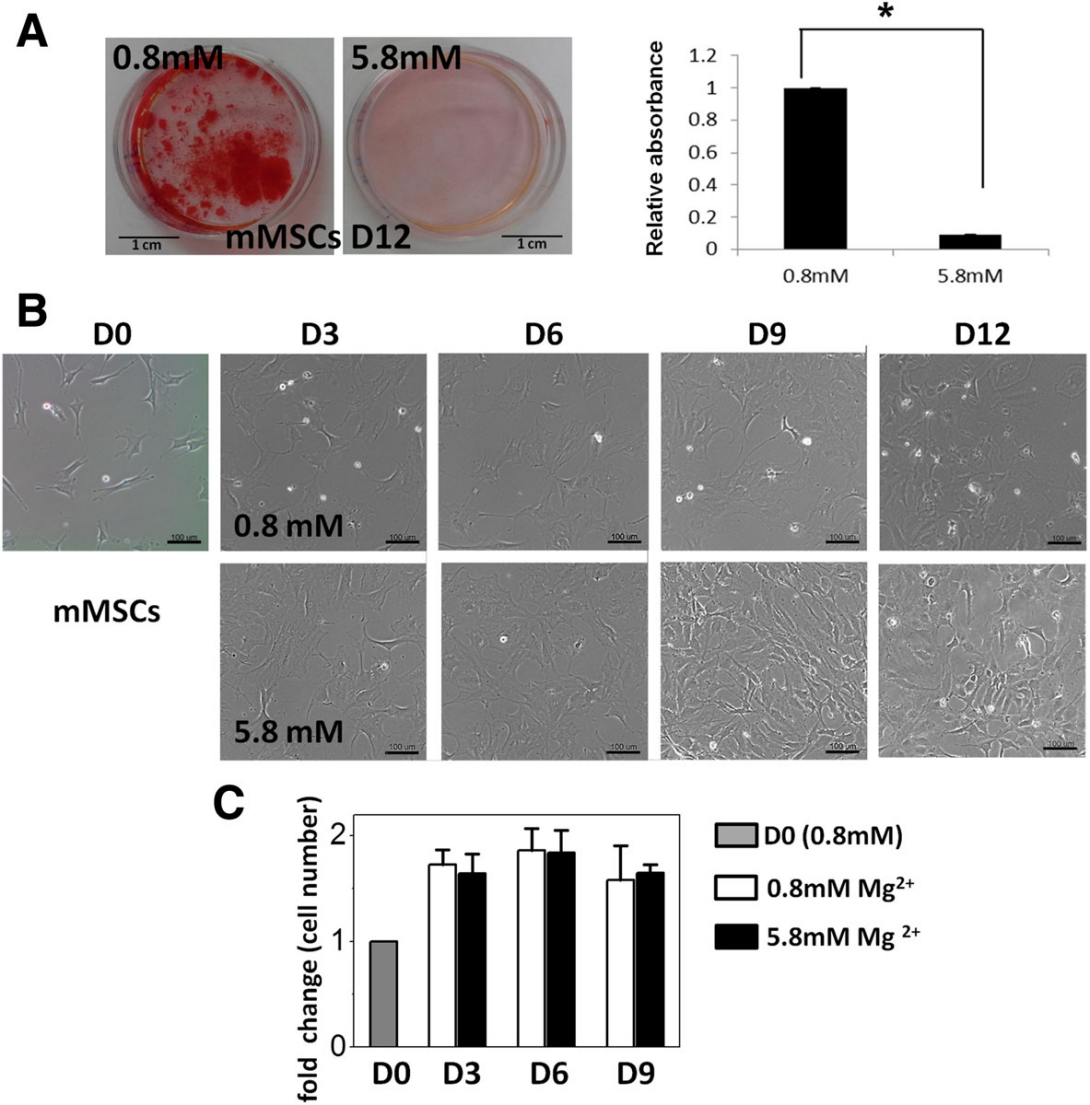
High concentration of extracellular magnesium inhibited mineralization of mouse MSCs during osteogenesis. **a** Alizarin Red S staining images (*left*) and quantification (*right*) of mouse MSCs (*mMSCs*) 12 days after osteogenic induction with normal (0.8 mM) and high (5.8 mM) extracellular magnesium concentration. Biological replicate *N* = 3 and technical replicate *n* = 3 for every biological replicate. **b** Images of mMSCs during osteogenic differentiation with normal and high extracellular magnesium concentration. *Scale bar*: 100 μm. **c** Cell number of differentiating mMSCs under osteogenic induction medium containing 0.8 and 5.8 mM magnesium for 0, 3, 6, and 9 days. Cell number was determined by counting the DAPI-positive cells (*N* = 3, *n* = 7) and normalized by cell number for day 0. Data presented as mean ± SEM ﻿(*﻿ *p* ﻿< 0.05). *D*: day

To study the effect of extracellular high concentration of magnesium on mineralization of MSCs during osteogenic differentiation, we supplied 5 mM MgCl_2_ in the osteogenic induction medium as the experimental group to make a total 5.8 mM magnesium concentration and employed Alizarin Red S staining to determine the presence of calcific deposition. Calcification results from mineral deposited by the extracellular matrix which is secreted during maturation of bone cells; therefore it is an important indicator for osteogenic differentiation. Our results showed that mMSCs, which were under osteogenic induction for 12 days, had significantly reduced calcium deposition when cultured in the medium with high magnesium concentration (5.8 mM) compared with those with physiological magnesium concentration (0.8 mM) (Fig. 1a). The morphologies of MSCs under 0.8 and 5.8 mM magnesium concentration both turned flat during osteogenic induction (Fig. 1b). In addition, there was no significant difference in the cell numbers of MSCs differentiated under 0.8 and 5.8 mM magnesium concentration (Fig. 1c).

**Fig. 2:**
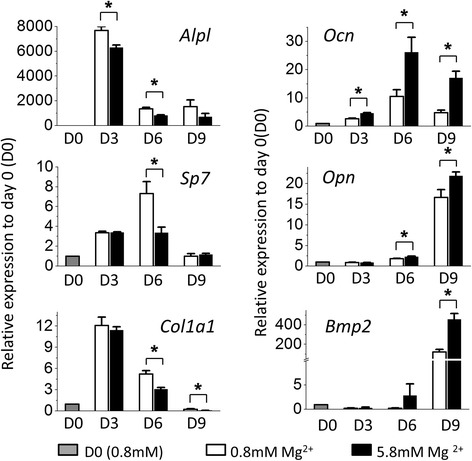
High concentration of extracellular magnesium modulated osteogenic differentiation of mMSCs. qPCR results of mMSCs under osteogenic differentiation for 0, 3, 6, and 9 days (*D0*, *D3*, *D6*, *D9*) with normal and high extracellular magnesium concentration. Relative gene expression indicates fold-change of gene expression in comparison with gene expression of undifferentiated MSCs on D0 (*N* = 3, *n* = 3), presented as mean ± SEM (* *p* ﻿< 0.05)﻿

We further investigated the effects of high extracellular magnesium on the expression of osteogenic marker genes of mMSCs. qPCR results showed that high concentration of magnesium significantly downregulated the gene expression of early markers including alkaline phosphatase (*Alpl*) on day 3 (*p* = 0.01) and day 6 (*p* = 0.016) after induction, osterix (*Sp7*) on day 6 (*p* = 0.039), and alpha-1 type I collagen (*Col1a1*) on day 6 (*p* = 0.017) and day 9 (*p* = 0.015) after the induction. On the contrary, high concentration of magnesium significantly upregulated gene expression of late markers including osteocalcin (*Ocn*) on day 3 (*p* < 0.001), day 6 (*p* = 0.049), and day 9 (*p* = 0.001) after induction, osteopontin (*Opn*) on day 6 (*p* = 0.048) and day 9 (*p* = 0.048) after induction, and bone morphogenetic protein 2 (*Bmp2*) on day 9 (*p* = 0.015) after induction (Fig. 2). Of note, the high fold increases of ALPL and BMP2 gene expression of differentiating MSCs compared with those of undifferentiated MSCs may be due to the extremely low expression of these two genes in undifferentiated cells (day 0).

Similarly, high magnesium concentration inhibited mineralization of hMSCs under osteogenic induction for 28 days. qPCR results showed that high concentration of magnesium significantly downregulated osterix (*Sp7*) gene expression on day 14 and upregulated osteonectin gene expression after day 7. Magnesium had no effect on gene expression of Runx2 and Col1A1. There is also no significant difference in cell numbers during osteogenic differentiation (more supporting data in Additional file 2: Figure S1A, S1B, S1C).

### *Slc41a1* knockdown promoted mineralization and attenuated magnesium inhibition

**Fig. 3:**
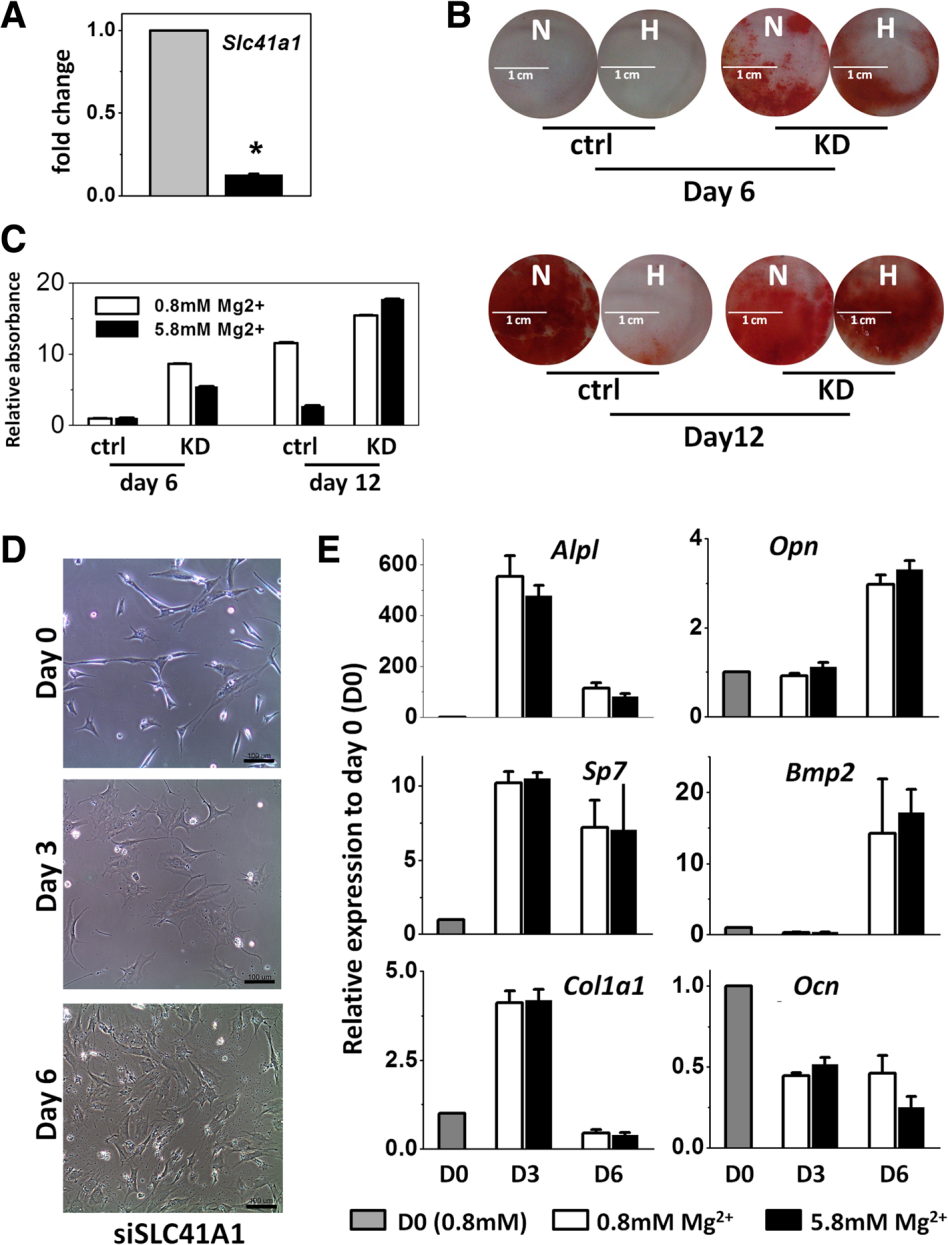
*Slc41a1* knockdown promoted mineralization and attenuated magnesium inhibition. **a** *Slc41a1* expression of undifferentiated mMSCs after *Slc41a1* gene knockdown (*N* = 3, *n* = 3). **b**, **c** Alizarin Red S staining (*left*) and the quantification (*right*) of wild-type mMSCs (*ctrl*) and mMSCs with *Slc41a1* gene knockdown (*KD*) 6 and 12 days after osteogenic induction with normal (*N*: 0.8 mM) and high (*H*: 5.8 mM) extracellular magnesium concentration (*N* = 3, *n* = 3). **d** Images of mMSCs with *Slc41a1* gene knockdown before and 3 and 6 days after osteogenic induction with normal magnesium concentration. *Scale bar*: 100 μm. **e** Gene expression of undifferentiated mMSCs with *Slc41a1* gene knockdown and 3 and 6 days after osteogenic induction (*D0*, *D3*, *D6*) under 0.8 and 5.8 mM magnesium concentration. Data normalized by the gene expression of undifferentiated *Slc41a1*-knockdown MSCs (*D0*) and presented as mean ± SEM (*N* = 3, *n* = 3) (* p< 0.05)

In order to identify the potential candidates that modulate the interaction of magnesium and MSCs during osteogenic differentiation, we initially screened several magnesium transporters, including SLC41A1, SLC41A2 as well as SLC41A3, and found that gene expression of SLC41A1 in differentiating MSCs was upregulated by extracellular high magnesium concentration. We hypothesized that SLC41A1 plays a role in magnesium homeostasis during osteogenic differentiation of MSCs and therefore silenced *Slc41a1* gene expression to study the effect of *Slc41a1* knockdown on mineralization of MSCs during osteogenic differentiation. Alizarin Red S staining demonstrated that treatment of siRNA targeting *Slc41a1* promoted calcification of mMSCs. The mMSCs with *Slc41a1* knockdown (KD-MSCs) exhibited calcific deposition as early as day 6 after osteogenic induction; whereas there was no calcification of the control group at the same day (Fig. 3a–c). Promotion of mineralization was also observed in human MSCs with *Slc41a1* knockdown (more supporting data in Additional file 2: Figure S1E). More interesting, even when cultured in osteogenic induction medium with high concentration of magnesium, KD-MSCs possessed similar potential for calcification as KD-MSCs cultured with physiological magnesium concentration (Fig. 3a–d). High magnesium concentration did not show significant inhibition on osteogenic differentiation of KD-MSCs. Regarding the osteogenic marker genes we tested, the levels of gene expression were similar between KD-MSCs cultured in physiological magnesium concentration and KD-MSCs in high magnesium concentration both 3 and 6 days after induction (Fig. 3e). Additionally, qPCR results revealed that even before the chemical induction (day 0), KD-MSCs were likely committed into an osteogenic lineage compared with wild-type MSCs (more supporting data in Additional file 2: Figure S2). Because KD-MSCs showed an extremely high degree of mineralization after 9 days of induction, we did not further perform qPCR assays on day 9.

### Gene expression and secreted MGP were elevated after *Slc41a1* was knocked down

**Fig. 4:**
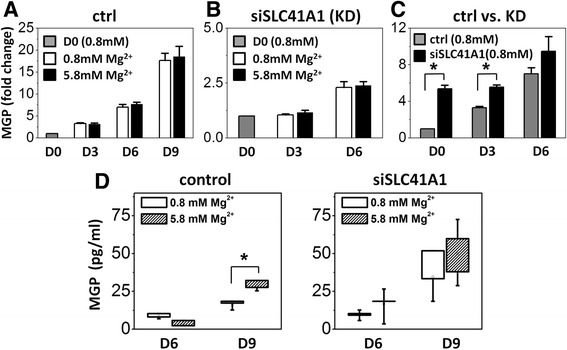
*Slc41a1* knockdown elevated MGP expression. **a**, **b** *Mgp* gene expression of control (*ctrl*) mMSCs and *Slc41a1*-knockdown mMSCs (*KD*) with normal and high extracellular magnesium concentration before and 3, 6, and 9 days after the osteogenic induction (*D0*, *D3*, *D6*, *D9*). Data normalized by the gene expression of undifferentiated ctrl and KD MSCs respectively (D0) (*N* = 3, *n* = 3). **c** *Mgp* gene expression of control and *Slc41a1* KD-mMSCs with normal extracellular magnesium concentration. Data normalized by *Mgp* gene expression of undifferentiated control MSCs (D0) (*N* = 3, *n* = 3). **d** Extracellular secreted MGP concentration obtained by ELISA assay (*N* = 3, *n* = 3). *MGP* matrix gla protein. * *p﻿*< 0.05﻿

Matrix gla protein (MGP) is known as a developmentally regulated modulator of mineralization. Our results showed that gene expression of *Mgp* increased with induction time during osteogenesis. Yet there was no significant difference between the groups cultured in normal and high magnesium concentration (Fig. 4a, b). The results demonstrated that magnesium concentration did not play a major role in regulating *Mgp* gene expression. We further investigated the relation of magnesium concentration and secreted MGP protein level. Even though gene expression of *Mgp* was significantly upregulated after *Slc41a1* was knocked down in the early stage of the osteogenic differentiation (Fig. 4c), the results of ELISA showed that secreted free MGP protein level did not reflect the mineralization ability between wild-type and *Slc41a1* knockdown groups 6 days after the induction, indicating that accumulated extracellular MGP protein was not high enough to affect mineralization on the 6th day and extracellular MGP protein was not the cause for the promotion or the inhibition of mineralization (Fig. 4d). After 9 days of osteogenic induction, only wild-type MSCs exhibited significant differences in secreted MGP level between normal and high magnesium concentration (*p* = 0.01). Because the affinity of MGP to HAP is sensitive to competitive HAP-binding protein and the surrounding ionic environment, magnesium may act as a competitor against MGP to reduce MGP–HAP binding and therefore increase the secreted unbound MGP level. Meanwhile, extracellular MGP protein secreted from *Slc41a1* KD-MSCs was relatively higher than that from wild-type MSCs, indicating that MGP protein of KD-MSCs was not utilized for HAP binding, possibly due to the matured mineralization of KD-MSCs, and most MGP was secreted in free form.

### SLC41A1 modulated Wnt/β-catenin anti-calcifying pathway

**Fig. 5:**
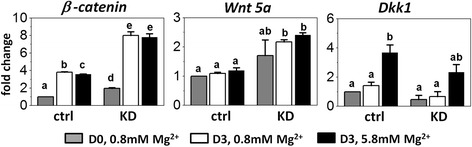
*Slc41a1* knockdown attenuated the high magnesium-induced inhibition of Wnt signaling. Gene expression of *Wnt5a*, *β-*c*atenin*, and *Dkk1* in control (*ctrl*) and *Slc41a1* knockdown (*KD*) mMSCs with normal and high extracellular magnesium concentration before and 3 days after osteogenic induction (*D0* and *D3*). Two-way analysis of variance followed by Tukey’s post-hoc tests was performed for multiple comparisons. *p* < 0.05 is defined statistically significant; *same letters* denote no significant differences. Data normalized by the gene expression of undifferentiated wild-type MSCs (D0) and presented as mean ± SEM (*N* = 3, *n* = 3)

Magnesium can inhibit Wnt/β-catenin activity and reverse calcification of vascular smooth muscle cells. Because our results showed that SLC41A1 mediated the magnesium regulation of mineralization during MSC osteogenic differentiation, we therefore hypothesized that SLC41A1 is involved in the Wnt/β-catenin anti-calcifying pathway. qPCR results showed that magnesium concentration did not have impact on gene expression of *Wnt5a* of wild-type MSCs after osteogenic induction, which is in the early stage of osteogenesis and before the onset of mineralization. However, high concentration of magnesium significantly downregulated *β-catenin* expression (*p* = 0.003) and the inhibition was diminished after the *Slc41a1* gene was knocked down. Dickkopf-1 (DKK1) is one of the secreted antagonists of the Wnt/β-catenin signaling pathway and its role in inhibiting mineralization has been well established. Interestingly, high concentration of magnesium significantly upregulated *Dkk1* gene expression (*p* = 0.018) and the upregulation was attenuated after *Slc41a1* gene knockdown (Fig. 5).

**Fig. 6:**
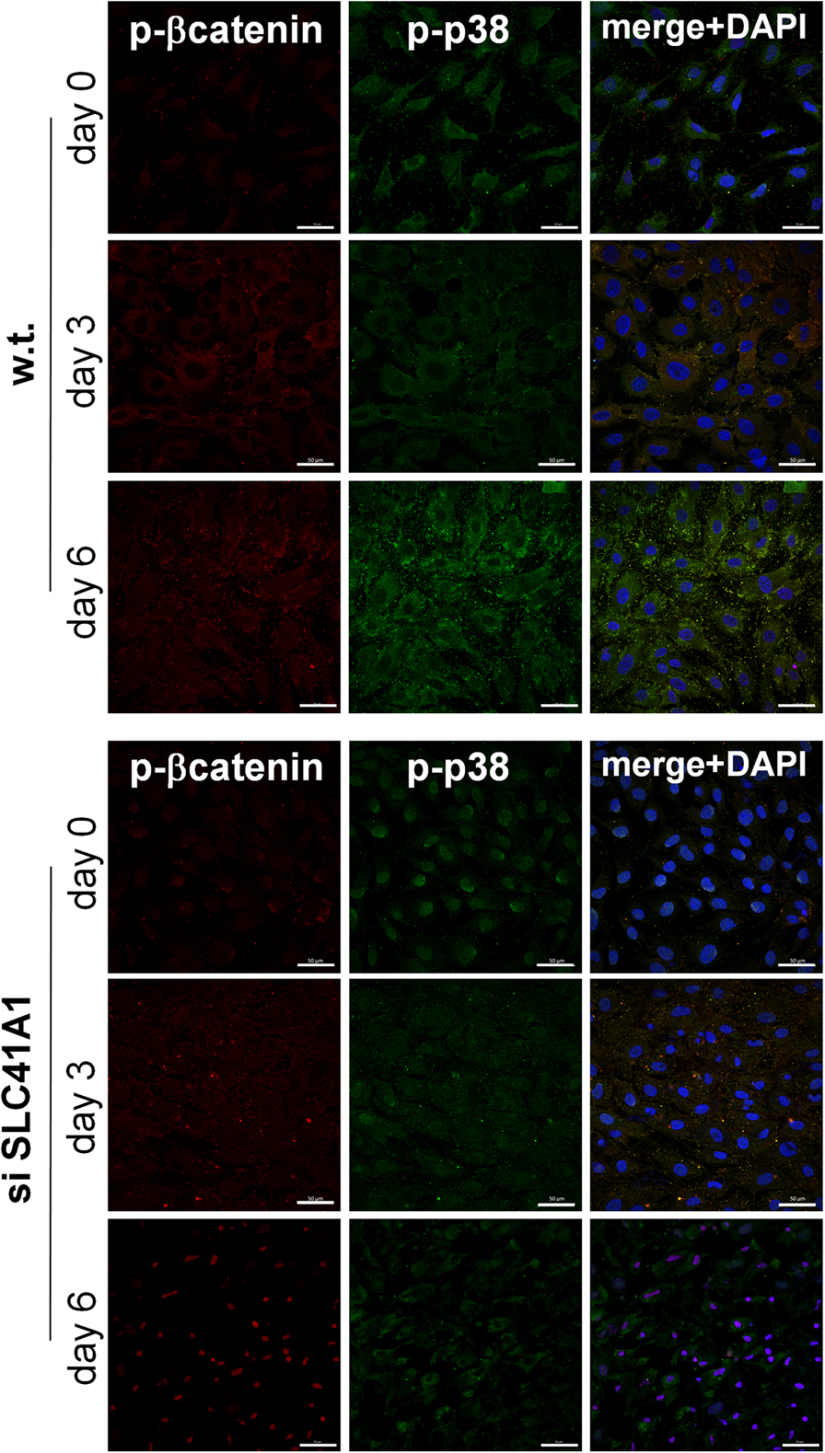
SLC41A1 modulated the localization of phosphorylated β-catenin. Immunofluorescent staining of phosphorylated β-catenin (*red*), phosphorylated p38 mitogen-activated protein kinases (*green*), and DAPI (*blue*) on wild-type (*w.t.*) MSCs and MSCs treated with siRNA (*si*) targeting SLC41A1 magnesium transporter. Staining was performed before osteogenic induction (day 0), as well as 3 and 6 days after the induction. *Scale bar*: 50 μm

Results of immunofluorescent staining showed that phosphorylated β-catenin localized in the nuclei of KD-MSCs 6 days after the induction; yet such nuclear localization of phosphorylated β-catenin was not observed in wild-type MSCs during this period of time. In addition, we also observed that phosphorylated p38 in KD-MSCs relocated to the cytoplasm from the nuclei on day 6 after the induction (Fig. 6). The results suggested that Wnt/β-catenin signaling is involved in SLC41A1-mediated osteogenic differentiation of MSCs.

## Discussion

A recent study showed that the *Slc41a1* gene expressed in mouse blood cells is downregulated immediately after exercise but returns to the basal level 24 hours after exercise [21]. The fact that the Mg^2+^ concentration in blood increases during short-term, high-intensity exercise [33] highlights the importance of the downregulation of this specific magnesium transport for maintaining proper physiological function. The study presented herein is the first report regarding the roles of SLC41A1 in mineralization during osteogenic differentiation. We suspect that Wnt signaling is one of SLC41A1-mediated regulation pathways for MSC osteogenic differentiation and the underlying mechanisms warrant further investigation. In addition, microarray data of MSCs in osteogenic induction medium with physiological magnesium concentration (0.8 mM) for 3 days showed that differentially expressed genes (DEGs) between wild-type MSCs and MSCs with *Slc41a1* gene knockdown were involved in focal adhesion and MAPK signaling pathways. Other DEGs of top-five hits were involved in the protein–protein interaction network of the podocyte as well as spinal cord injury (more supporting data in Additional file 2: Figure S3). The MAPK signaling pathway, focal adhesion, and Akt-mTOR signaling pathway are known to play roles in modulating osteogenic differentiation [34–37]. Whether these pathways are affected by SLC41A1-mediated magnesium ion directly or by SLC41A1-kinase activity will be investigated in the future.

Matrix gla protein (MGP) is a developmentally regulated modulator of mineralization. Constitutive MGP expression in the chick limb has been shown to block intramembranous and endochondral ossification [38]. Mice lacking MGP die soon after birth due to the massive calcification of arterial and cartilages [39]. Although it may have other unknown effects on ectopic calcification, MGP has been suggested as a potent inhibitor of HAP crystal growth during mineralization process [40]. In addition to the competitive role of magnesium against MGP binding HAP crystal, previous study also demonstrated that high extracellular magnesium inhibits excess calcium-induced mineralization and excess calcium-induced MGP expression in prechondrogenic cell line ATDC5. Such inhibitions by excess magnesium are suggested to be mediated by inhibiting the expression of calcium-sensing receptor (CaSR) [41]. Another study also indicated that the inhibition of mineralization by high concentration of extracellular Mg^2+^ may be through the modulation of calcium oscillations via suppression of spontaneous ATP release and inactivation of purinergic receptors [42]. Similarly, we also observed decreased intracellular calcium concentration as well as decreased calcium influx when mMSCs were cultured in osteogenic induction medium with high concentration magnesium after 9 days (more supporting data in Additional file 2: Figure S4). This phenomenon could be explained by the competition between calcium and magnesium ions for the same transporter, such as transient receptor potential cation channel, subfamily M, member 7 (TRPM7). TRPM7 is permeable to a number of divalent metal ions, including Mg^2+^ and Ca^2+^; it is mechanosensitive and important in osteogenesis in MSCs [43]. The results presented in our study demonstrated that SLC41A1 is important for the regulation of magnesium during mineralization; however, we cannot exclude the roles that TRPM7 and other magnesium/calcium transporters may play during MSC mineralization.

High extracellular magnesium inhibits not only mineralized matrix deposition by MSCs [42] and osteoblasts [44], but also inhibits the excess calcium-promoted mineralization in prechondrogenic cells [41]. On the other hand, it has been proposed that deficiency in magnesium causes osteoporosis. Insufficient intake of magnesium reduces the secretion of parathyroid hormone and vitamin D, and leads to hypocalcaemia as well as the disturbance of calcium homeostasis [10]. Because parathyroid hormone also regulates bone turnover rates and the homeostasis of the related components, such as calcium and magnesium, it is not surprising that biochemical reactions involved in magnesium and bone metabolisms are complicated. Some studies showed that supplement of magnesium in daily diets could alleviate the rate of bone loss. On the other hand, our study suggests that caution should be taken when magnesium supplementation is used as a therapy for bone mass loss because high magnesium could lead to mineralization impairment.

## Conclusion

High concentration of extracellular magnesium modulates gene expression of MSCs during osteogenic differentiation and inhibits the mineralization process. Additionally, we identified magnesium transporter SLC41A1 that regulates the interaction of magnesium and MSCs during osteogenic differentiation. Knockdown of SLC41A1 promotes the mineralization during osteogenesis and Wnt signaling is suggested to be involved in the SLC41A1-mediated regulation. Together, tissue-specific SLC41A1 could be a potential treatment for bone mass loss; in addition, caution should be taken regarding the role of magnesium in osteoporosis and the design of magnesium alloys for implantation because local high magnesium concentration could generate from fast degrading.

## Additional files

Additional file 1: Table S1. Presenting primer sequences of mMSCs used for real-time PCR. (DOC 55 kb)
Additional file 2:
**Figure S1** showing that high concentration of extracellular magnesium inhibited mineralization of human MSCs during osteogenesis, **Figure S2** showing relative expressions of osteogenic marker genes of mouse MSCs with *Slc41a1*gene knockdown as well as of differentiation wild-type MSCs compared with those of the undifferentiated and magnesium-untreated (0.8 mM magnesium) wild-type MSCs, **Figure S3** showing the top-five pathways in which DEGs between wild-type and *Slc41a1*-knockdown MSCs were involved, and **Figure S4** showing that high extracellular magnesium concentration decreased intracellular calcium concentration of mouse MSCs 6 days after osteogenic induction. (DOC 1139 kb)

## Abbreviations

ALPL: Alkaline phosphatase
BMP2: Bone morphogenetic protein 2
COL1A1: Alpha-1 type I collagen
DKK1: Dickkopf WNT signaling pathway inhibitor 1
HAP: Hydroxyapatite
MGP: Matrix gla protein
MSC: Mesenchymal stem/stromal cell
OCN: Osteocalcin
OPN: Osteopontin
SLC41A1: Solute carrier family 41 member 1
SP7: Osterix
TRPM7: Transient receptor potential cation channel, subfamily M, member 7
