# Supplementary material for: Knockdown of SLC41A1 magnesium transporter promotes mineralization and attenuates magnesium inhibition during osteogenesis of mesenchymal stromal cells
Source: Stem Cell Res Ther. 2017 Feb 21;8:39. doi: 10.1186/s13287-017-0497-2 (PMC5320718; doi:10.1186/s13287-017-0497-2)
Supplement: Additional file 1: Table S1. — Presenting primer sequences of mMSCs used for real-time PCR. (DOC 55 kb) [file 13287_2017_497_MOESM1_ESM.doc]

**Knockdown of SLC41A1 magnesium transporter promotes mineralization and attenuates the magnesium inhibition during the osteogenesis of mesenchymal stromal cells**

Yu-Tzu Tsao, Ya-Yi Shih, Yu-An Liu, Yi-Shiuan Liu, Oscar K. Lee

**Supplemental Table**

## Table S1 Sequences of RT-PCR primers for mouse MSCs

| **gene** | **accession number** | **sequences** | | **product length**  **(nt)** | | **annealing temperature (^o^C)** | |
| --- | --- | --- | --- | --- | --- | --- | --- |
| Rps18 | ENSMUSG00000008668.6 | | cttccacaggaggcctacac / tggtgttgagtactcgcaaaat | | 96 | | 59 / 59 |
| Osterix | ENSMUSG00000060284.5 | | tgcttcccaatcctatttgc / agctcagggggaatcgag | | 66 | | 60 / 60 |
| Osteocalcin | ENSMUSG00000074483.2 | | agactccggcgctacctt / ctcgtcacaagcagggttaag | | 93 | | 59 / 59 |
| Col1a1 | ENSMUSG00000001506.10 | | catgttcagctttgtggacct / gcagctgacttcagggatgt | | 96 | | 59 / 59 |
| Alpl | ENSMUSG00000028766.4 | | ttaagggccagctacaccac / agggacctgagcgttggt | | 107 | | 60 / 60 |
| Bmp2 | ENSMUSG00000027358.6 | | cggactgcggtctcctaa / ggggaagcagcaacactaga | | 70 | | 59 / 60 |
| Osteopontin | ENSMUSG00000029304 | | ggaggaaaccagccaagg / tgccagaatcagtcactttcac | | 107 | | 60 / 60 |
| Mgp | NM_008597.3 | | ccgagacaccatgaagagc / gattcgtagcacagggttgc | | 75 | | 59 / 60 |
| β Catenin | NM_001165902.1 | | tgcagatcttggactggaca / aagaacggtagctgggatca | | 77 | | 60 / 59 |
| Wnt 5a | ENSMUSG00000021994.8 | | atgaagcaggccgtaggac / cttctccttgagggcatcg | | 140 | | 60 / 60 |
| Dkk1 | NM_010051.3 | | ccgggaactactgcaaaaat / ccaaggttttcaatgatgctt | | 94 | | 59 / 59 |
| Slc41a1 | NM_173865.3 | | gatgatcacGgggaaTatgg / ggatccagccaaagacga | | 92 | | 59 / 59 |
