# Supplementary material for: Knockdown of SLC41A1 magnesium transporter promotes mineralization and attenuates magnesium inhibition during osteogenesis of mesenchymal stromal cells
Source: Stem Cell Res Ther. 2017 Feb 21;8:39. doi: 10.1186/s13287-017-0497-2 (PMC5320718; doi:10.1186/s13287-017-0497-2)
Supplement: Additional file 2: — Figure S1 showing that high concentration of extracellular magnesium inhibited mineralization of human MSCs during osteogenesis, Figure S2 showing relative expressions of osteogenic marker genes of mouse MSCs with Slc41a1gene knockdown as well as of differentiation wild-type MSCs compared with those of the undifferentiated and magnesium-untreated (0.8 mM magnesium) wild-type MSCs, Figure S3 showing the top-five pathways in which DEGs between wild-type and Slc41a1-knockdown MSCs were involved, and Figure S4 showing that high extracellular magnesium concentration decreased intracellular calcium concentration of mouse MSCs 6 days after osteogenic induction. (DOC 1139 kb) [file 13287_2017_497_MOESM2_ESM.doc]

**Knockdown of SLC41A1 magnesium transporter promotes mineralization and attenuates the magnesium inhibition during the osteogenesis of mesenchymal stromal cells**

Yu-Tzu Tsao, Ya-Yi Shih, Yu-An Liu, Yi-Shiuan Liu, Oscar K. Lee

**Supplemental Figures**

**Figure S1**

**
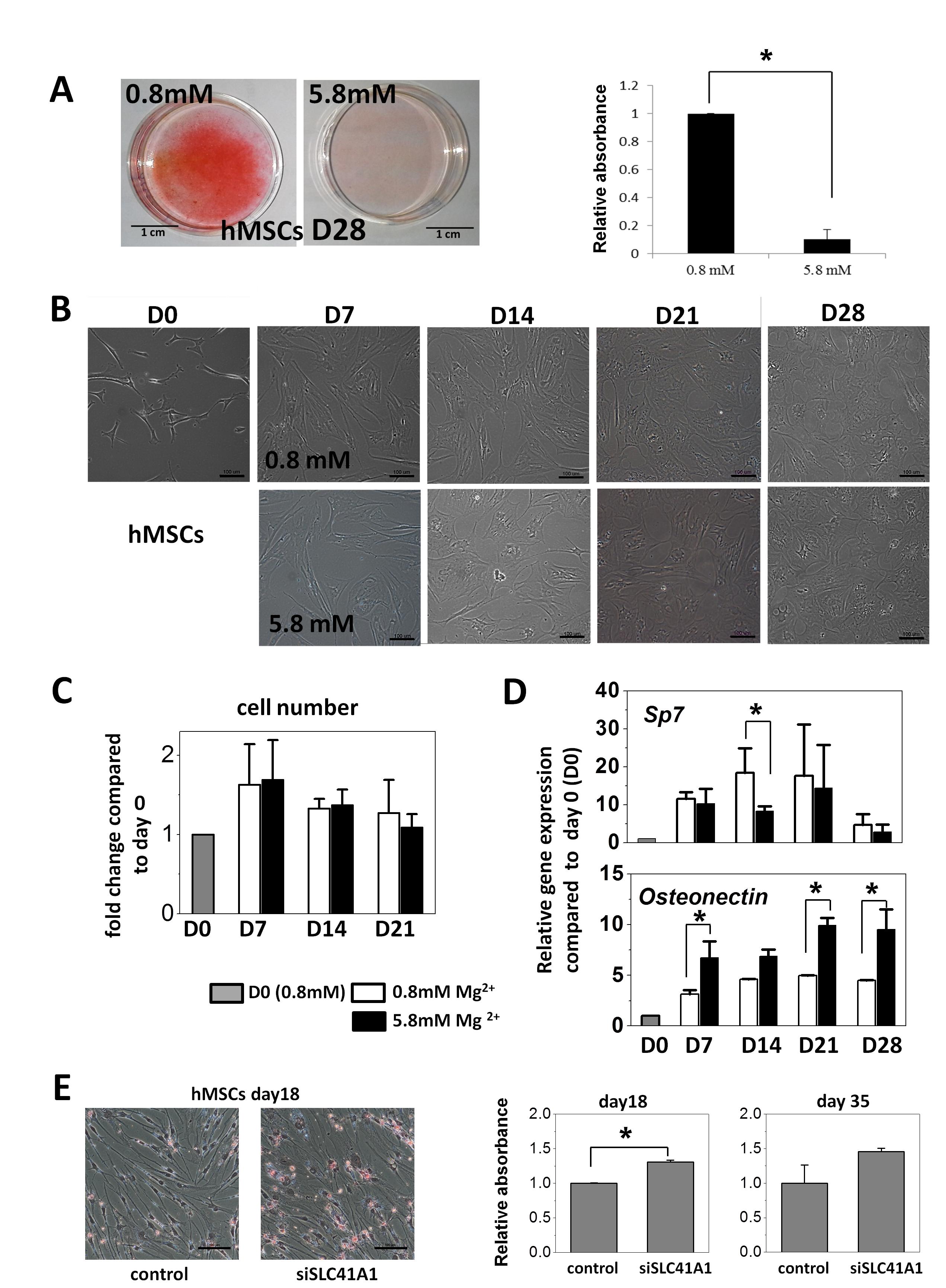
**

**Figure S1.** High concentration of extracellular magnesium inhibited mineralization of human MSCs during osteogenesis. **(A)** Alizarin red S staining and quantitation of human MSCs (hMSCs) 28 days after osteogenic induction with normal (0.8mM) and high (5.8mM) extracellular magnesium concentration. (N=3, n=3) **(B)** Images of hMSCs during osteogenic differentiation with normal and high extracellular magnesium concentration. Scale bar: 100 μm. **(C)** Cell number of differentiating hMSCs under osteogenic inductiom medium containing 0.8mM and 5.8mM magnesium for 0, 7, 14, and 21 days. Cell number was determined by counting the DAPI positive cells (N=3, n=7) and normalized by cell number of day 0. **(D)** Relative gene expressions of *Osterix* (*Sp7*) and *Osteonectin.* (N=3, n=3) **(E)** Alizarin red S staining (left) and the quantitation (right) of control and si*SLC41A1*-treated hMSCs 18 days after osteogenic induction with normal (0.8mM) extracellular magnesium concentration. Note that cells were seeded at 15000 cells per cm^2^, not 4000 cells per cm^2^, for osteogenic induction in this specific experiment. All data were represented as mean ± SEM. Scale bar: 100 μm. Biological replicate N = 3 and technical replicate n = 3 for every biological replicate.

**Figure S2**

**A**

**
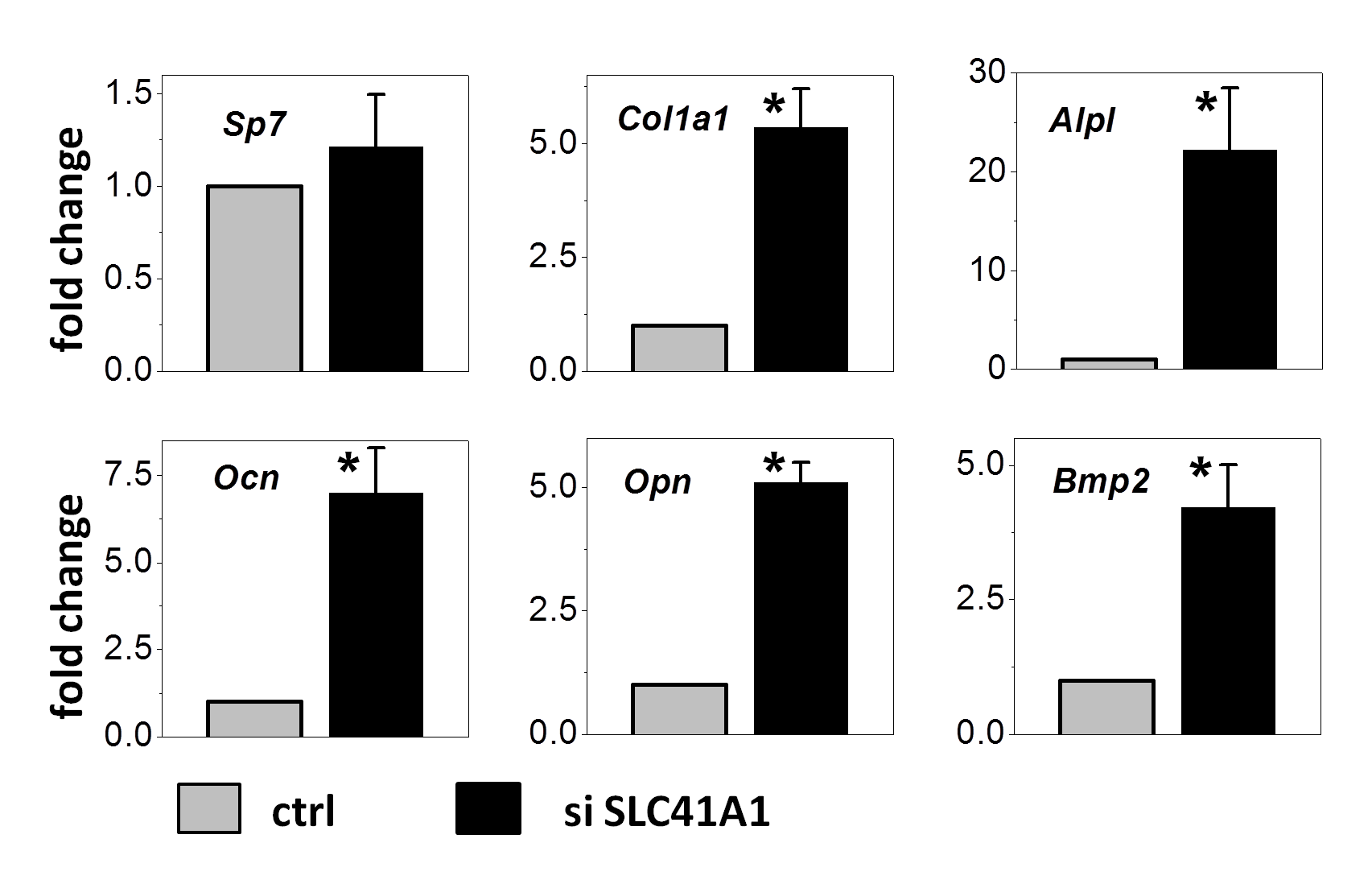
**

B

|  | **Ctrl day 0** | **KD day 0** | **Ctrl day 3 0.8mM** | **Ctrl day 3 5.8mM** | **KD day 3 0.8mM** | **KD day 3 5.8mM** | **Ctrl day 6 0.8mM** | **Ctrl day 6 5.8mM** | **KD day 6 0.8mM** | **KD day 6 5.8mM** |
| --- | --- | --- | --- | --- | --- | --- | --- | --- | --- | --- |
| **Sp7** | 1 | 1.2 | 3.4 | 3.4 | 12.4 | 12.7 | 7.3 | 3.4 | 8.7 | 8.5 |
| **Col1a1** | 1 | 5.4 | 12.1 | 11.4 | 22 | 22.4 | 5.2 | 3.1 | 2.4 | 2.1 |
| **Alpl** | 1 | 22.2 | 7691.3 | 6311.6 | 12300.8 | 10615 | 1348.8 | 824.5 | 2539.3 | 1780.4 |
| **Ocn** | 1 | 7.0 | 2.7 | 4.6 | 3.1 | 3.6 | 10.6 | 26.2 | 3.2 | 1.7 |
| **Opn** | 1 | 5.1 | 0.9 | 0.9 | 4.7 | 5.7 | 1.8 | 2.3 | 15.2 | 16.9 |
| **Bmp2** | 1 | 4.2 | 0.2 | 0.3 | 1.4 | 1.5 | 0.2 | 2.8 | 60 | 72.3 |

**Figure S2.** **(A)** Gene expressions of undifferentiated mMSCs (day 0, D0) with *Slc41a1*gene knockdown under 0.8mM magnesium concentration compared to those of wild-type mMSCs. Data were represented as mean ± SEM. * indicated p-value < 0.05 as statistically different. **(B)** Relative expressions (fold changes) of osteogenic marker genes of mMSCs with *Slc41a1*gene knockdown (KD) as well as differentiation wild-type mMSCs (Ctrl) compared to those of the undifferentiated and magnesium-untreated (0.8mM magnesium) wild-type mMSCs (Ctrl day 0).

**Figure S3**


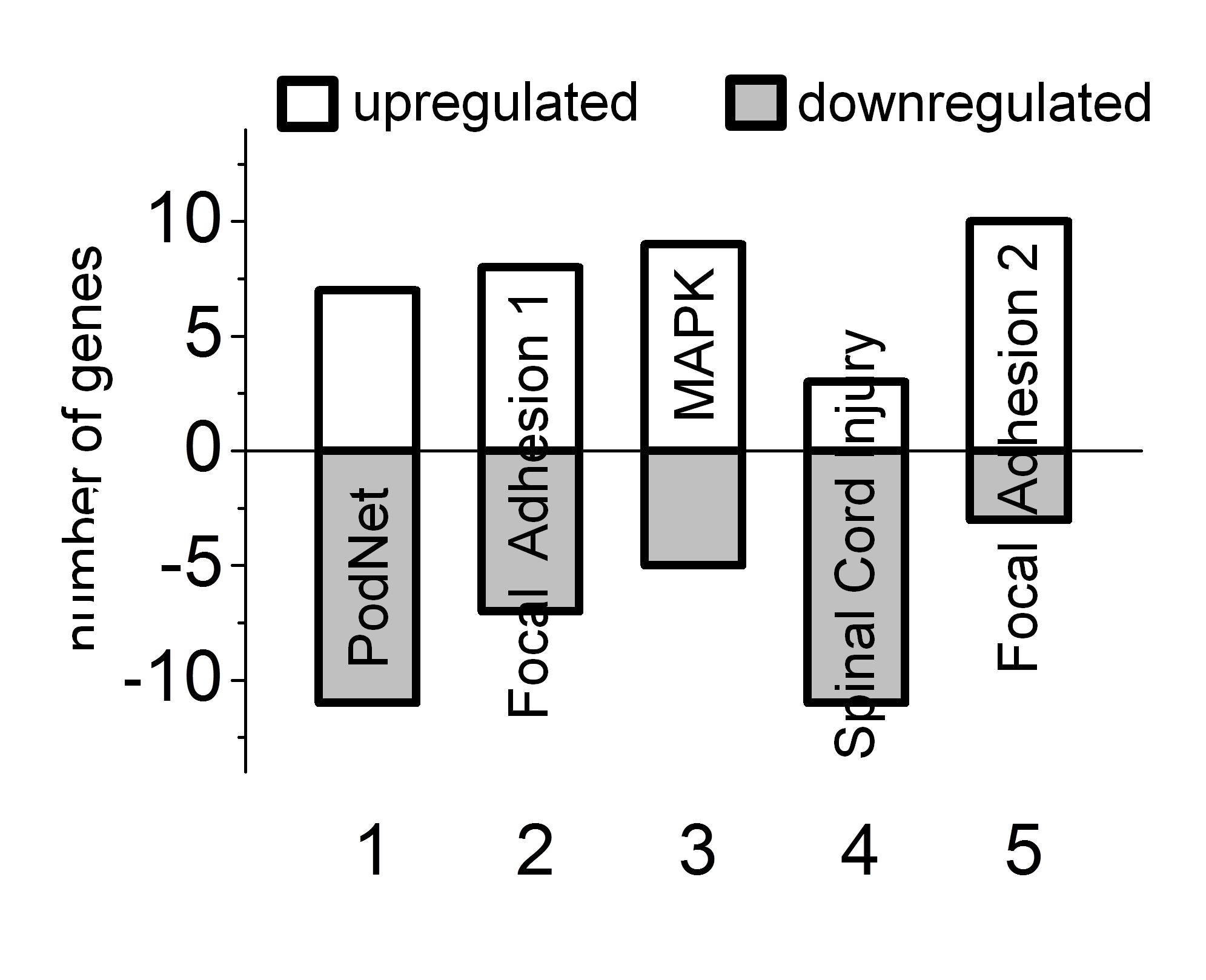


**Figure S3.** Top five pathways that differentially expressed genes (DEGs) between wild-type and *Slc41a1*-knockdown MSCs were involved. Samples were collected 3 days after osteogenic induction. RMA files generated by Affymetrix Expression Console from Microarray data (Mouse430_2) were analyzed by Transcriptome Analysis Console (TAC) 3.0 (Affymetrix Inc.) with filter set as default (2 fold change). 1: Protein-protein interactions in the podocyte (PodNet), 2: Focal adhesion, 3: MAPK signaling pathway, 4: Spinal cord injury, 5: Focal adhesion-PI3K-Akt-mTOR-signaling pathway.

**Figure S4**


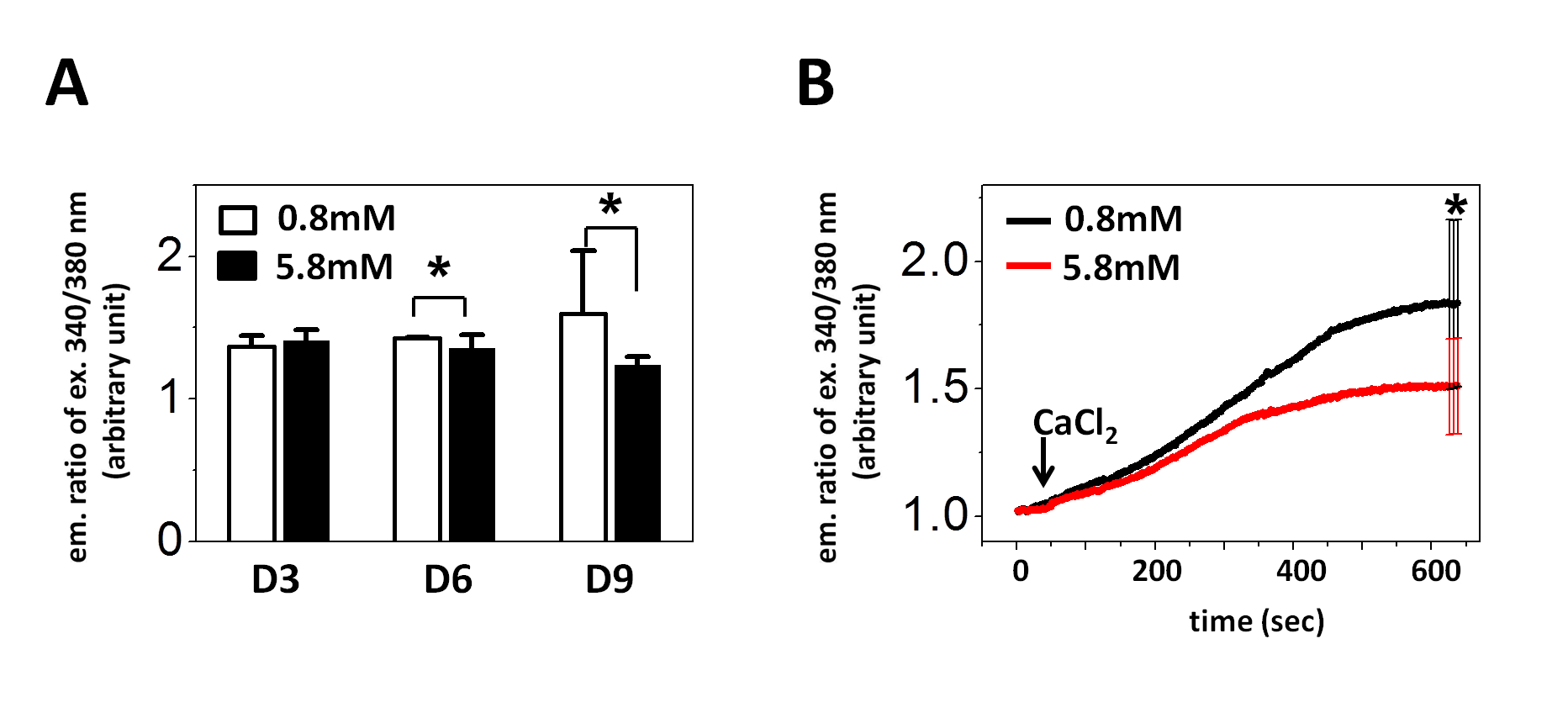


**Figure S4.** (A) High extracellular magnesium concentration decreased intracellular calcium concentration of mMSCs 6 days after osteogenic induction. N=3, n=22. Fura-2 AM (Sigma-Aldrich) was used to estimate the intracellular free calcium. Cells were incubated in serum-free LG-DMEM with Fura dye for 1 hour at 37^o^C, and then washed by Fura-free LG-DMEM twice. Ratiometric measurements (510nm emission by 340nm/380nm excitation) were performed using calcium imaging system (Zeiss Axio Observer with Lambda DG4) and data were analyzed by AxioVision 4 Physiology Module. (B) High extracellular magnesium concentration decreased calcium influx induced by 2mM CaCl2. mMSCs were osteogeneic induced for 9 days. n =18. Data were represented as mean ± SD. * indicated p-value < 0.05 as statistically different.
